# Supplementary material for: Structural basis of specific lysine transport by Pseudomonas aeruginosa permease LysP
Source: Nat Commun. 2025 Dec 4;17:37. doi: 10.1038/s41467-025-66618-7 (PMC12764776; doi:10.1038/s41467-025-66618-7)
Supplement: Supplementary file 1 — Supplementary Information [file 41467_2025_66618_MOESM1_ESM.pdf]

Supplementary Information

Structural basis of specific lysine transport by *Pseudomonas aeruginosa* permease LysP

Deniz Bicer[#,1,2,3,4,†], Rei Matsuoka[#,5], Aurélien F. A. Moumbock[1,2,3,6], Preethi Sukumar[7], Albert Suades[8], Harish Cheruvara[9], Andrew Quigley[9], David Drew[8], Els Pardon[10,11], Jan Steyaert[10,11], Peter J.F. Henderson[7], Martin Caffrey[12], Julia J. Griese[4] & Emmanuel Nji[1,2,3,13,14,*]

[1]BioStruct-Africa, Nairobi, Kenya

[2]BioStruct-Africa, Kumasi, Ghana

[3]BioStruct-Africa, Stockholm, Sweden.

[4]Department of Cell and Molecular Biology, Uppsala University, 751 24 Uppsala, Sweden.

[5]OMass Therapeutics Ltd, Building 4000, Chancellor Court, John Smith Drive, ARC Oxford, OX4 2GX, UK.

[6]Institute of Pharmaceutical Sciences, Albert-Ludwigs-Universität Freiburg, Freiburg, Germany.

[7]Astbury Centre for Structural Molecular Biology, University of Leeds, Leeds LS2 9JT, UK.

[8]Department of Biochemistry and Biophysics, Science for Life Laboratory, Stockholm University, Stockholm, Sweden.

[9]Membrane Protein Laboratory, Diamond Light Source Ltd., Research Complex at Harwell, Didcot OX11 0DE, UK.

[10]VIB-VUB Center for Structural Biology, VIB, Pleinlaan 2, 1050 Brussels, Belgium.

[11]Structural Biology Brussels, Vrije Universiteit Brussel, Pleinlaan 2, 1050 Brussels, Belgium.

[12]Schools of Medicine and Biochemistry & Immunology, Trinity College, Dublin D02 R590, Ireland.

[13]Department of Parasitology and Microbiology, Centre for Research in Infectious Diseases, P.O. Box 13591, Yaoundé, Cameroon.

[14]Visiting Research Fellow, Membrane Protein Laboratory, Diamond Light Source Ltd., Research Complex at Harwell, Didcot OX11 0DE, UK.

[#]Contributed equally.

[†]Present address: Department of Molecular Biology and Genetics, Aarhus University, Universitetsbyen 81, 8000 Aarhus, Denmark.

*Correspondence: emmanuel.nji@biostructafrica.org

This PDF file includes:

Supplementary Figures 1-7

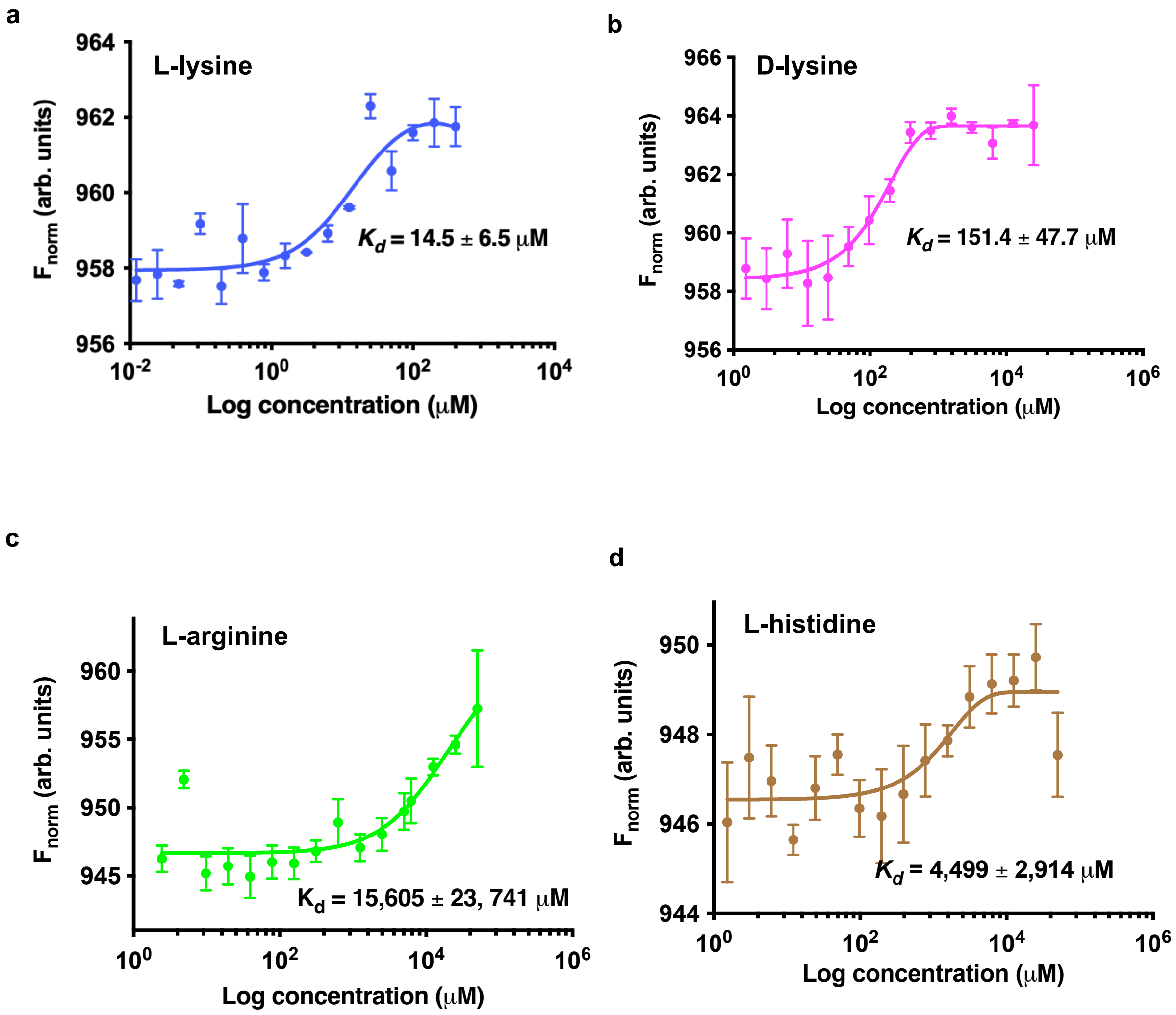

Supplementary Figure 1. Functional characterization of LysP a, Microscale thermophoresis (MST) binding profile of LysP to L-lysine with a K_d of 14.5 μM. **b**, MST binding profile of LysP to D-lysine with a K_d of 151 μM. **c**, MST binding profile of LysP to L-arginine with a K_d of 15.6 mM. **d**, MST binding profile of LysP to L-histidine with a K_d of 4.5 mM. Error bars represent the mean ± s.e.m. of four independent experiments. a.u. stands for arbitrary units. Source data are provided as a Source Data file.

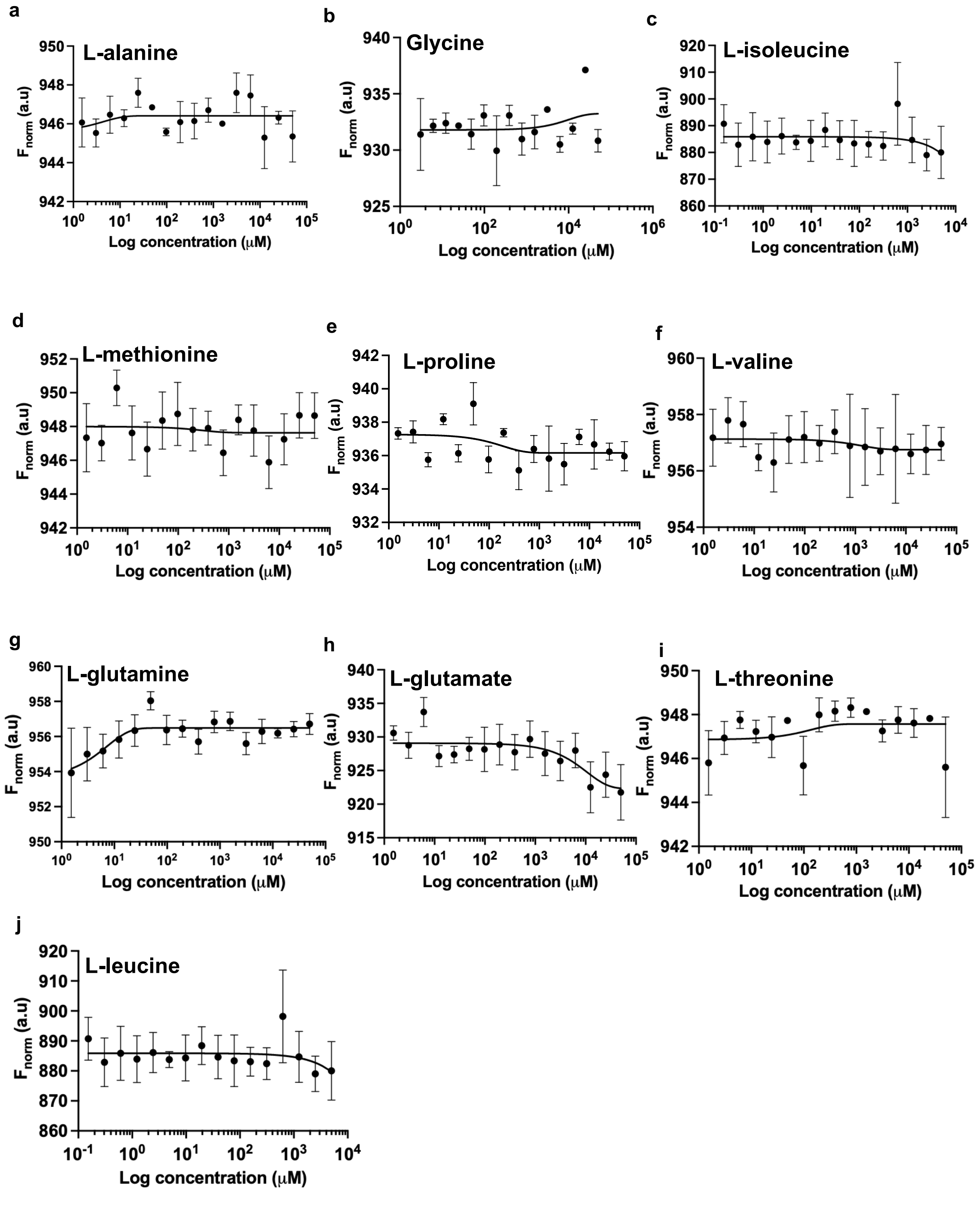

Supplementary Figure 2. Microscale thermophoresis (MST) analysis of LysP interaction with various amino acids, showing no detectable binding. a, L-Alanine; **b,** Glycine; **c,** L-Isoleucine; **d,** L-Methionine; **e,** L-Proline; **f,** L-Valine; **g,** L-Glutamine; **h,** L-Glutamate; **i,** L-Threonine; **j,** L-Leucine. Error bars represent the mean ± s.e.m. from two to three independent experiments. a.u. stands for arbitrary units. Source data are provided as a Source Data file.

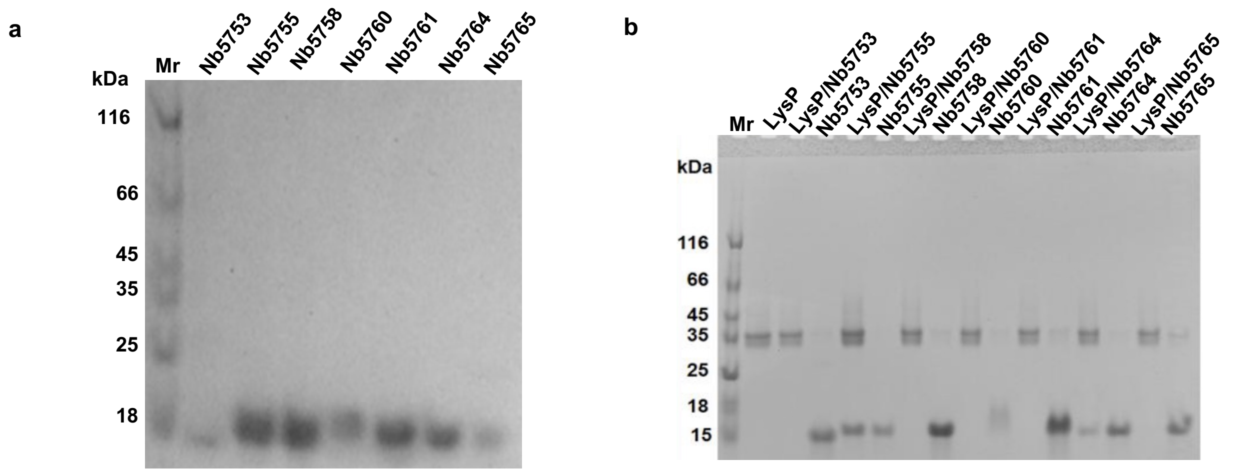

Supplementary Figure 3. Purification and screening of nanobody binders against LysP without added L-lysine. **a,** SDS PAGE analysis of purified nanobodies (Nb5753, Nb5755, Nb5758, Nb5760, Nb5761, Nb5764, and Nb5765). Mr = molecular weight marker. **b**, SDS-PAGE analysis of size-exclusion chromatography peaks of nanobody-LysP complexes. Mr = molecular weight marker. Source data are provided in the Source Data file.

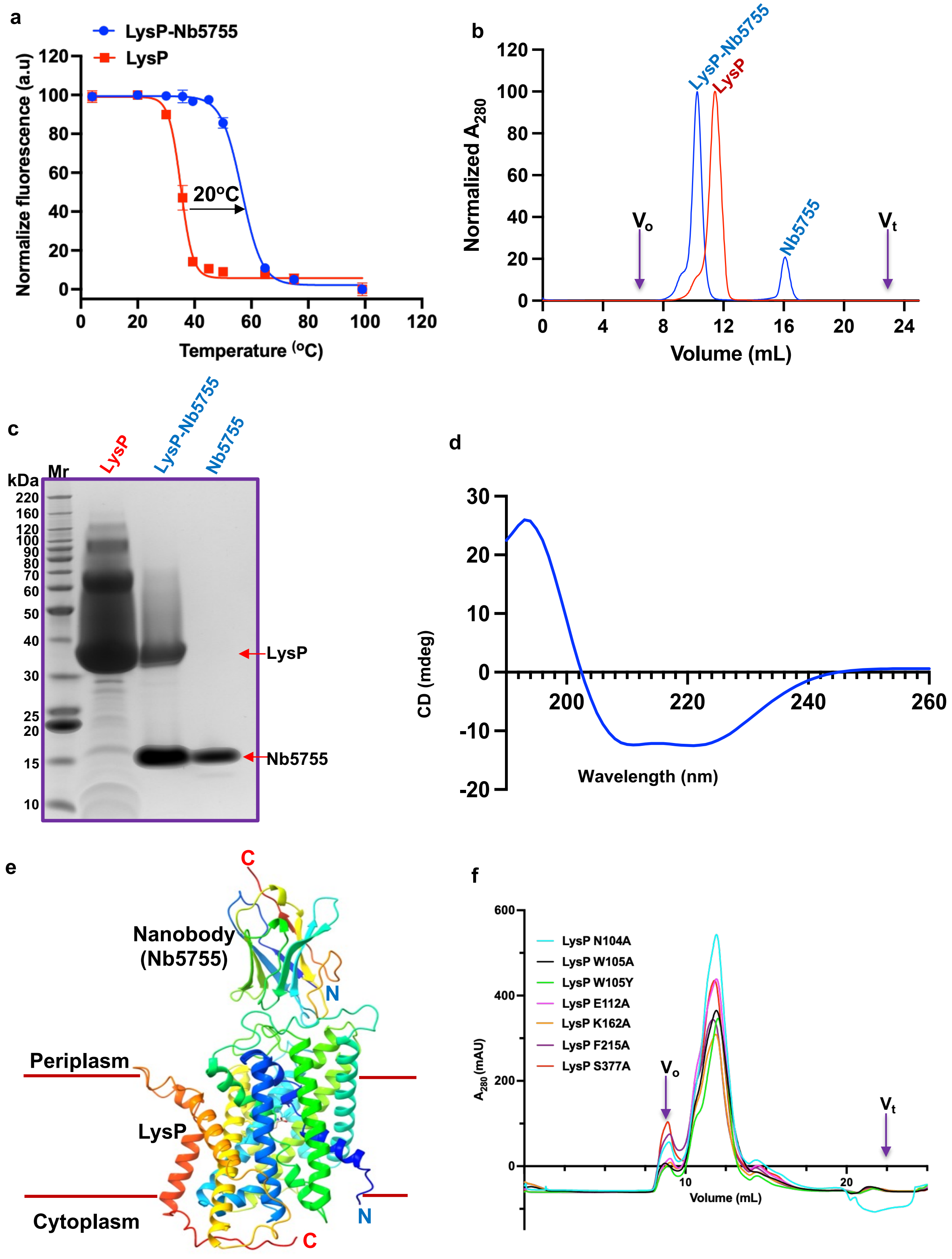

Supplementary Figure 4. Biochemical and structural characterization of LysP. **a,** Normalized Green Fluorescent Protein Thermal Shift (GFP-TS) melting curve of LysP-GFP with Nb5755 (blue) and without

Nb5755 (red). Error bars represent the mean ± s.d. of three independent titrations. a.u. stands for arbitrary units. **b,** Gel filtration chromatogram of LysP (red) and LysP-Nb5755 complex (blue). The small peak at a V_e of 16 mL represents excess free Nb5755 (blue). n=1 for a single experiment **c,** SDS PAGE analysis of the peaks shown in **b**, lane 1 (free LysP), lane 2 (LysP-Nb5755 complex) and lane 3 (free Nb5755) and Mr (molecular weight standards). **d,** Circular dichroism spectrum of free LysP. n=3 for three independent experiments. **e,** Cryo-EM structure of the LysP–L-lysine–NbCA5755 complex viewed from the membrane plane. **f,** Gel filtration chromatograms of the various LysP mutants. V_o denotes the void volume, and V_t the included volume. Source data are provided as a Source Data file.

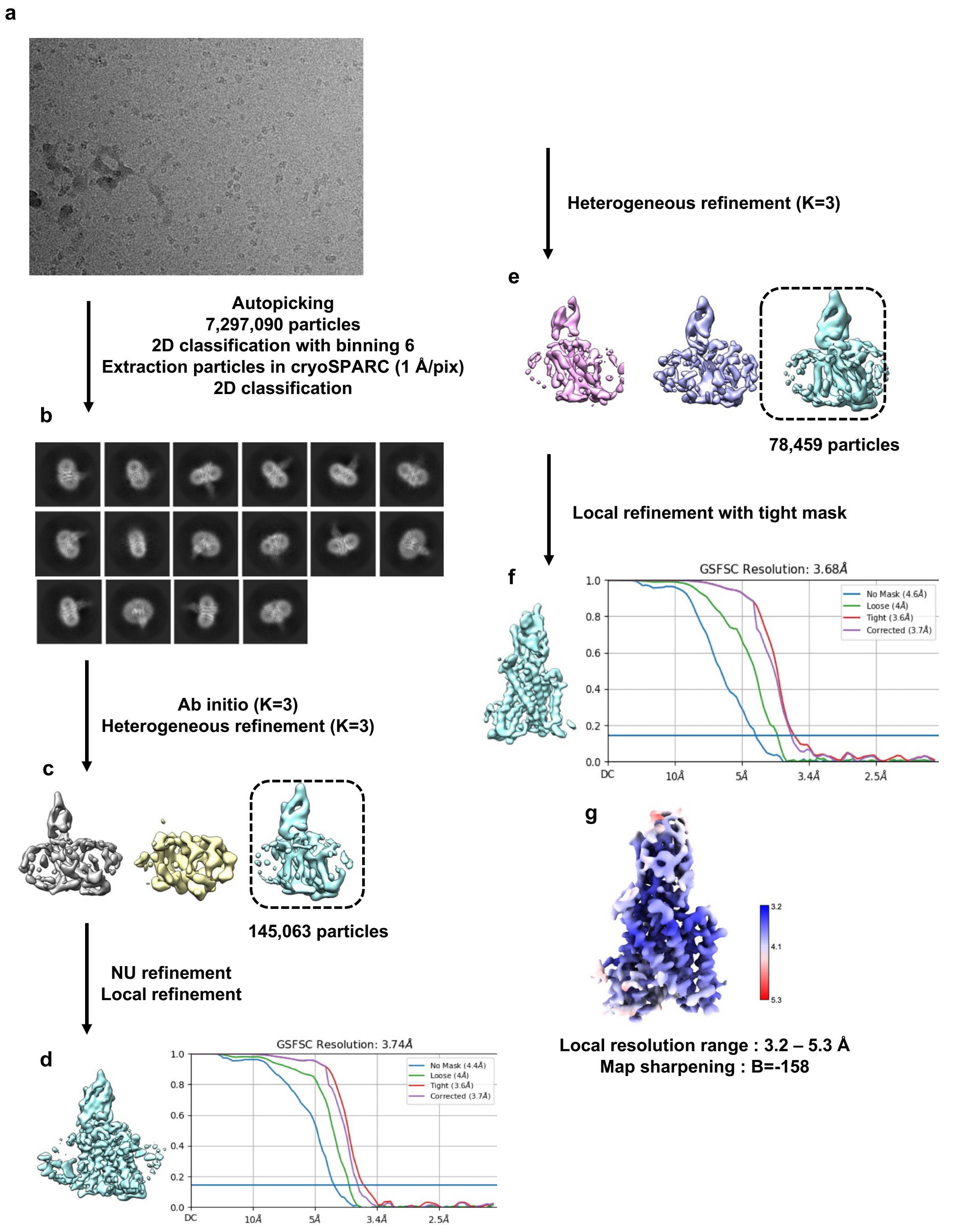

Supplementary Figure 5. Cryo-EM data processing workflow **a**, Representative EM micrograph. 10,427 micrographs were collected, and 7,297,090 particles were picked by blob picking. **b**, The selected 2D

class-averages. **c**, 3D classes from heterogenous refinement and selected 145,063 particles for further processing. **d**, The first non-uniform refinement 3D map that reached 3.74 Å at the gold standard FSC value of 0.143. **e**, Heterogeneous refinement with different low-pass filtered map (20 Å or 40 Å**)**. **f**, The local refinement using tight mask without micelle region. The final resolution reached to 3.68 Å at the gold standard FSC. **g**, Colored map according to local resolution estimation.

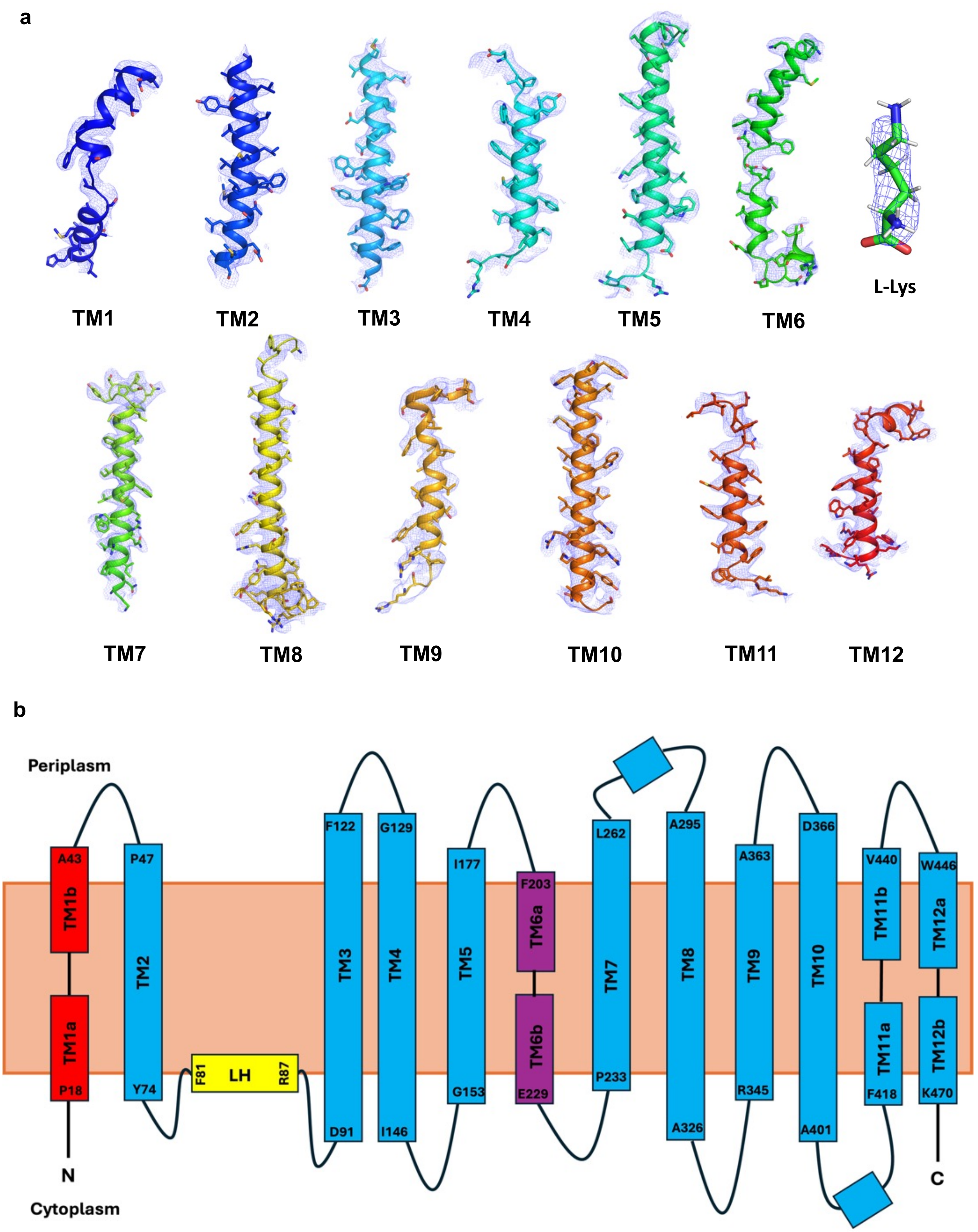

Supplementary Figure 6. Cryo-EM maps and model-to-map fit of LysP a, Cryo-EM density corresponding to representative individual transmembrane helices of LysP and L-lysine at σ = 7.0. **b**,

Topology of the cryo-EM structure of LysP. LysP has 12 transmembrane helices with both C- and N-termini located in the cytoplasm. Helices 1, 6, 11 and 12 are broken in the middle, Helices 2 and 3 are linked by a lateral cytoplasmic helix and helices 10 and 11 are linked by a hydrogen bond turn followed by a bend.

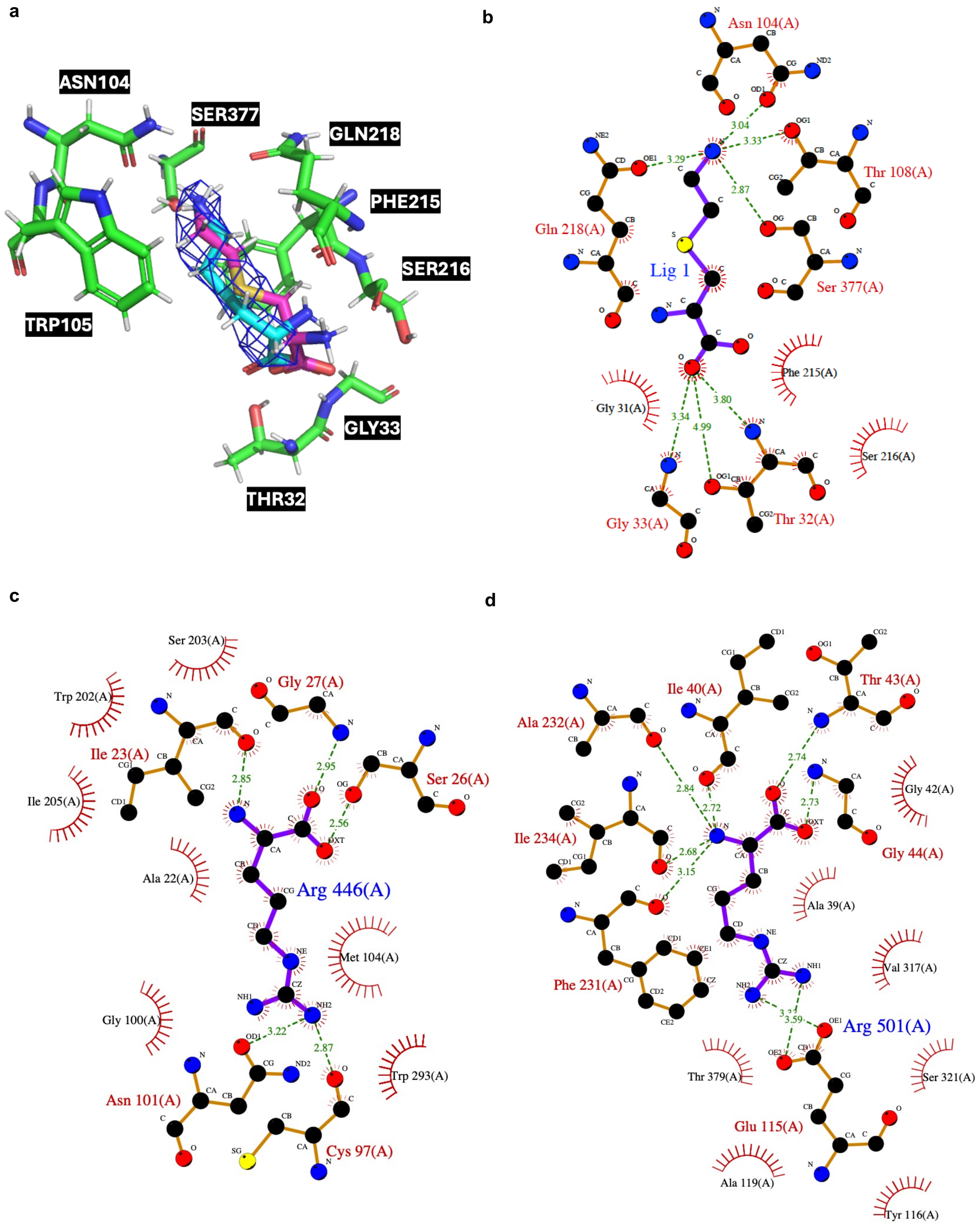

Supplementary Figure 7. Comparison of the binding site residues in LysP, *E. coli* AdiC and G. *kaustophilus* ApcT. **a,** LysP binding site with L-lysine (cyan) and docked L-4-thialysine (magenta) overlaid on the electron density map of L-lysine. **b,** LigPlot showing coordinating residues in the L-4-thialysine docked into the LysP structure. **c,** LigPlot showing the residues coordinating L-arginine in *E. coli* AdiC. **d,** LigPlot showing the residues coordinating L-arginine in *G. kaustophilus* ApcT.
