# Supplementary material for: Structural basis of specific lysine transport by Pseudomonas aeruginosa permease LysP
Source: Nat Commun. 2025 Dec 4;17:37. doi: 10.1038/s41467-025-66618-7 (PMC12764776; doi:10.1038/s41467-025-66618-7)
Supplement: Supplementary file 2 — Description of Additional Supplementary Files [file 41467_2025_66618_MOESM2_ESM.pdf]

Description of Additional Supplementary Files

File name: Supplementary Movie 1
Description: Conformational changes of LysP during L-Lysine binding and transport revealed by Normal Mode Analysis.
